# Impacts of El Niño Southern Oscillation and Indian Ocean Dipole on dengue incidence in Bangladesh

**DOI:** 10.1038/srep16105

**Published:** 2015-11-05

**Authors:** Shahera Banu, Yuming Guo, Wenbiao Hu, Pat Dale, John S. Mackenzie, Kerrie Mengersen, Shilu Tong

**Affiliations:** 1 School of Public Health and Social Work, Queensland University of Technology, Brisbane, QLD 4059, Australia; 2 School of Population Health, University of Queensland, Brisbane, QLD 4006, Australia.; 3 Environmental Futures Research Institute, Griffith School of Environment, Griffith University, Brisbane, QLD 4111, Australia; 4 Faculty of Health Sciences, Curtin University, GPO Box U1987, Perth, WA 6845, Australia; 5 School of Mathematical Sciences and Institute for Future Environments, Queensland University of Technology, Brisbane, QLD 4059, Australia

## Abstract

Dengue dynamics are driven by complex interactions between hosts, vectors and viruses that are influenced by environmental and climatic factors. Several studies examined the role of El Niño Southern Oscillation (ENSO) in dengue incidence. However, the role of Indian Ocean Dipole (IOD), a coupled ocean atmosphere phenomenon in the Indian Ocean, which controls the summer monsoon rainfall in the Indian region, remains unexplored. Here, we examined the effects of ENSO and IOD on dengue incidence in Bangladesh. According to the wavelet coherence analysis, there was a very weak association between ENSO, IOD and dengue incidence, but a highly significant coherence between dengue incidence and local climate variables (temperature and rainfall). However, a distributed lag nonlinear model (DLNM) revealed that the association between dengue incidence and ENSO or IOD were comparatively stronger after adjustment for local climate variables, seasonality and trend. The estimated effects were nonlinear for both ENSO and IOD with higher relative risks at higher ENSO and IOD. The weak association between ENSO, IOD and dengue incidence might be driven by the stronger effects of local climate variables such as temperature and rainfall. Further research is required to disentangle these effects.

Dengue is a major public health problem in many countries of the tropical and subtropical regions of the world. Dengue viruses that cause the disease are transmitted through the mosquito vectors *Aedes aegypti* (primary vector) and *Ae. albopictus* (secondary vector)^1^. *Ae. aegypti* prefers to lay its eggs in artificial containers commonly found in and around homes. Containers used for water storage are important in producing large numbers of adult mosquitoes in close proximity to humans^1^. It is well established that the transmission of dengue viruses can be influenced by several factors including environmental and climatic factors, host-pathogen interactions and population immunological factors^1^. Dengue is sensitive to climatic conditions as its mosquito vector requires standing water to breed and warm ambient temperature for larval development and virus replication^2^,^3^. Temperature influences the life cycle of *Aedes* mosquitoes including growth rate and larval survival and the length of reproductive cycle. Higher temperatures decrease the length of viral incubation within the vector, and thus increase the chance of mosquitoes becoming infective in their life span. Adult mosquito survival and biting activity are related to humidity while rainfall provides an increased number of breeding habitats^2^,^3^. Some studies have reported that the El Niño Southern Oscillation (ENSO) plays an important role in the inter-annual variation of dengue transmission^4^,^5^. ENSO is the fluctuation in atmospheric pressure and sea surface temperature (SST) in the equatorial Pacific Ocean. ENSO is a contributor to monsoon rainfall in the Indian region and influences world weather^6^. The large positive values of ENSO are associated with El Niño events which cause dry conditions in the Indian region^7^. Conversely, La Niña events are associated with large negative values of ENSO that cause increased rainfall and wet conditions in this region. Variability in the cholera incidence in Dhaka, Bangladesh between 1980 and 1998 was associated with ENSO^8^,^9^. However, the relationship between ENSO and dengue in Bangladesh has not been rigorously analysed.

The Indian Ocean Dipole (IOD) is a coupled ocean-atmosphere phenomenon in the Indian Ocean, which is characterized by anomalous cooling of SST in the south eastern equatorial Indian Ocean and anomalous warming of SST in the western equatorial Indian Ocean^10^. During a positive IOD event the East African region receives above normal rainfall, while rainfall is reduced in Indonesia and in Australia causing drought^11^. The IOD also plays an important role as a modulator of the Indian summer monsoon rainfall (ISMR) and influences the correlation between the ISMR and ENSO^12^. A positive IOD event significantly increases the monsoon rainfall over the Indian region and reduces the effects of ENSO on ISMR^12^. There is evidence that rainfall influenced dengue patterns in Bangladesh^13^,^14^. However, the effect of IOD on dengue incidence has not previously been examined. Clarifying the effects of IOD could assist in the development of an appropriate early warning system for dengue epidemics and aid in disease control. Here, we examined the relationship between ENSO, IOD and the incidence of dengue, using a 13-years dataset from Bangladesh where the disease is endemic^13^,^15^.

## Results

### Summary statistics

**Figure 1:**
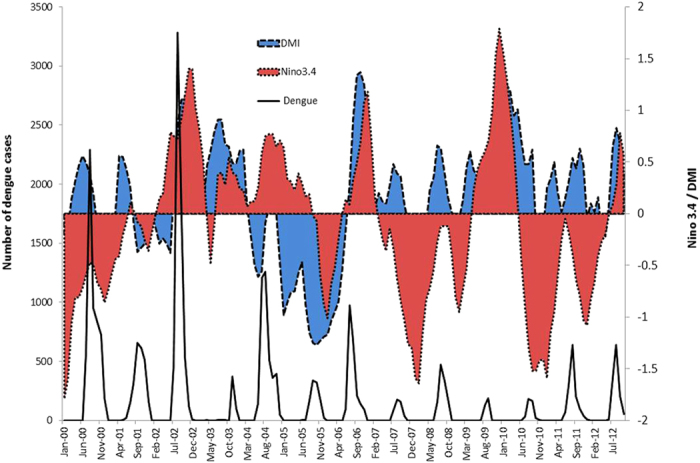
Time series for the number of dengue cases each month in Bangladesh, Nino3.4 (SST in the Nino3.4 region), and the DMI.

The ENSO index used in this study is the Nino3.4. Nino3.4 is the difference between monthly average SST for the area between 5^o^ N, 5 ^o^S, 120 ^o^W and 170 ^o^W and the average for that value for the years 1981–2000. ENSO events are defined as 5 consecutive overlapping 3-month periods at or above the +0.5^o^ SST anomaly for warm (El Niño) events and at or below the −0.5 anomaly for cold (La Niña) events. The threshold is further broken down into weak (with a 0.5 to 0.9 SST anomaly), moderate (1.0 to 1.4) and strong ( ≥1.5) events. We observed moderate El Niño events in 2002–2003 and 2009–2010 (Fig. 1). A weak El Niño event occurred in 2006–2007. During 2007–2008, a moderate La Niña event occurred while a strong La Niña event occurred in 2010–2011(Fig. 1). The Dipole Mode Index (DMI) is the difference in SST between the western equatorial Indian Ocean (50 ^o^E–70 ^o^E and 10 ^o^S–10 ^o^N) and the south eastern equatorial Indian Ocean (90 ^o^E–110 ^o^E and 10 ^o^S–0 ^o^N) which provides an index for IOD. Positive IOD events occurred in 2002 and 2006 during which the DMI peaked in October. During 2010 IOD event, the DMI peaked in February (Fig. 1).

**Figure 2:**
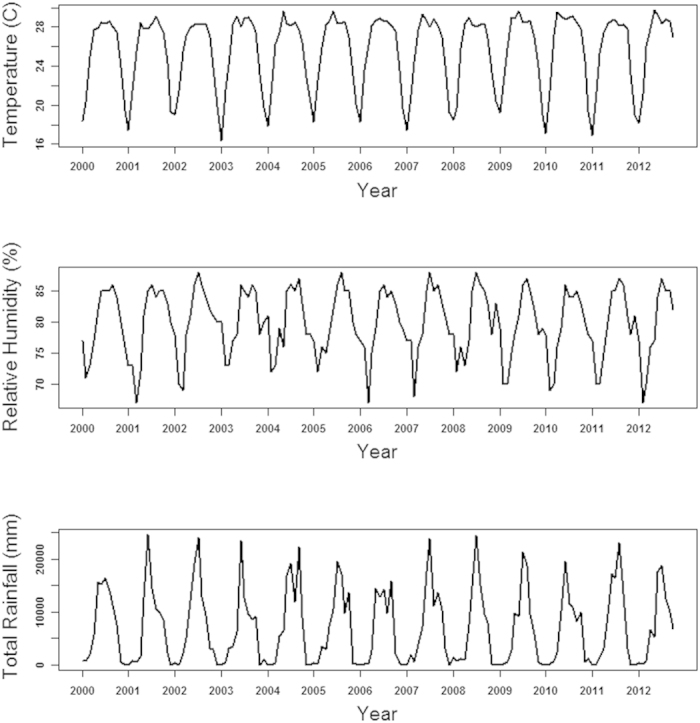
Monthly time series for temperature, humidity and total rainfall in Bangladesh.

We used the monthly dengue incidence rate per 100,000 populations in Bangladesh from January 2000 to October 2012 (Table 1). The mean monthly dengue incidence rate was 0.13 per 100, 000 populations (Table 1). The monthly mean of temperature, rainfall and relative humidity were 25.5 ^o^C, 7086 mm and 79.85%, respectively, between 2000 and 2012 in Bangladesh (Table 1). The monthly mean of Nino 3.4 and DMI were −0.1 and 0.12, respectively (Table 1). Figure 2 shows the monthly time series for temperature, humidity and total rainfall in Bangladesh. We calculated the partial correlation coefficient between dengue incidence rate and climate variables by controlling the effects of other climate variables. When we calculated the correlation between two climate variables, we accounted for the effects of other climate variables. The two tailed significance level for the correlation at *p* < 0.01 was calculated. Partial correlation coefficients between climate variables and dengue incidence rate show that only humidity was significantly correlated (r = 0.26, *p* < 0.01) with dengue incidence rate after controlling for other covariates including temperature, rainfall, DMI and Nino3.4 (Table 2). The Nino3.4 was significantly (r = 0.29, *p* < 0.01) associated with DMI after controlling for temperature, rainfall and humidity (Table 2). The partial correlation coefficient between rainfall and temperature was 0.57 (*p* < 0.01) during the study period (Table 2). The correlation between rainfall and humidity was also significant (r = 0.7, *p* < 0.01).

### Coherence between ENSO, IOD and dengue

**Figure 3:**
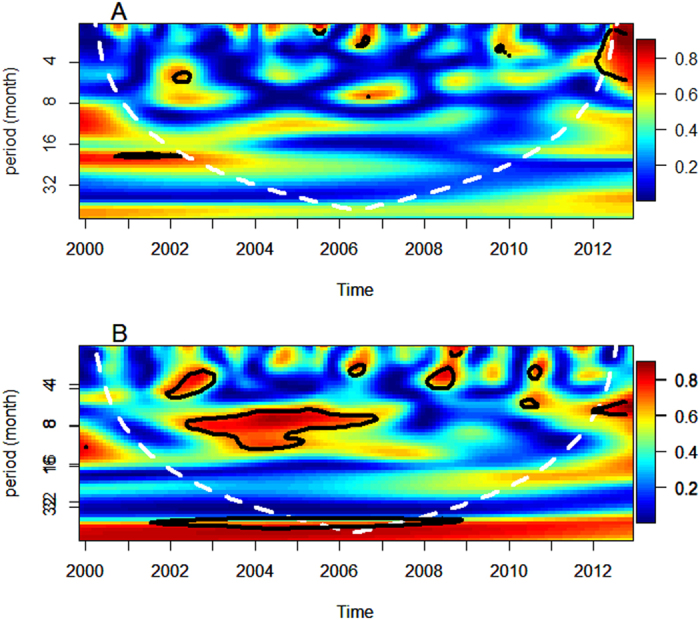
Squared coherence between Nino3.4, DMI and dengue incidence rate in Bangladesh (A: Nino3.4 and dengue, B: DMI and dengue). Statistical significance for coherence is determined by Monte Carlo simulations (300).Blue, low coherence; red, high coherence. The black lines show the areas where coherence is significantly high (95% confidence level). The cone of influence (white curve) indicates the region not influenced by edge effects.

We used wavelet coherence analysis to determine whether the presence of a particular frequency at a given time in dengue incidence corresponded to the presence of the same frequency climate covariates such as Nino3.4 and DMI. The results show that there were coherent cycles between dengue incidence rate and Nino3.4 in the 1–2 year band from 2001 to 2008 (Fig. 3A). The wavelet coherency between Nino3.4 and the dengue incidence rate was greater than 0.4 for most times in the 1–2 year band; however, it did not reach statistical significance. The coherence between dengue and DMI was significant in the 1 year band during the 2002 to 2007 (Fig. 3B).We also performed wavelet coherency between local climate variables (temperature and rainfall) and Nino3.4 (Supplementary Figure S4). The results show that there was moderate coherency between Nino3.4 and temperature in the 1–2 year band when rainfall was not significantly coherent in annual cycle. There was significant coherency between temperature, rainfall and DMI in the 1-year band (Supplementary Figure S5).

### The effects of ENSO and IOD on dengue

**Figure 4:**
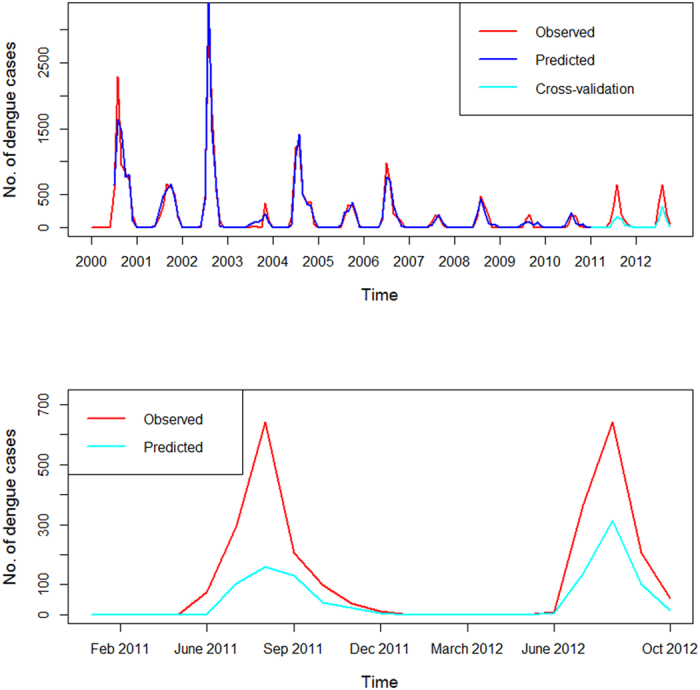
Cross validation of distributed lag nonlinear model. A natural cubic spline with three degrees of freedom was used for both Nino3.4 and DMI to smooth the data.

**Figure 5:**
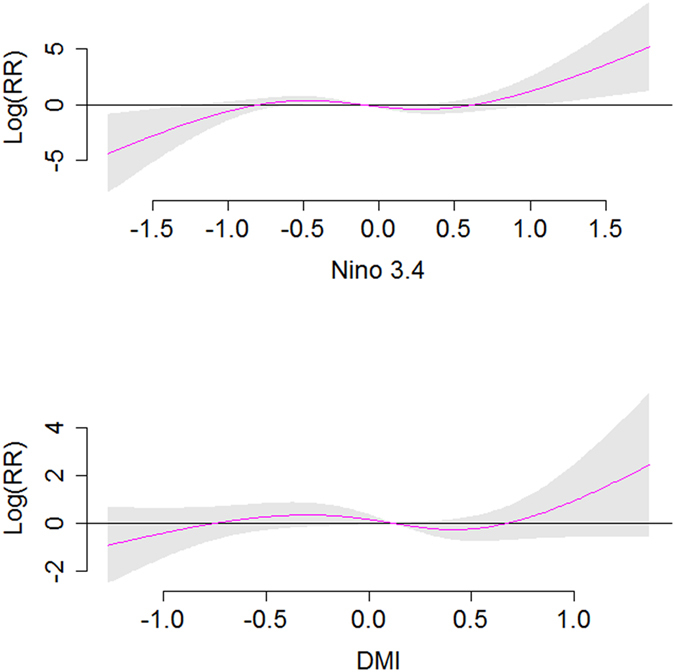
Estimated overall effects of ENSO/ IOD on dengue incidence rate after adjustment of potential confounders. The centre line in each graph represents the estimated spline curve, and the shadow represents 95% CI.

A Poisson time series model combined with the distributed lag nonlinear model (DLNM) was used to estimate the effects of Nino3.4 and DMI on dengue incidence^16^,^17^. According to the DLNM, Nino3.4 explained 56% (adjusted R^2^ = 0.562) variation in the dengue incidence, when DMI explained 77% (adjusted R^2^ = 0.77) variation (Table 3). Our best fit model included Nino 3.4, DMI, temperature, humidity and rainfall, which explained more than 90% (adjusted R^2^ = 0.929) variation in dengue incidence (Table 3). The model that included only local climate variables (temperature, humidity and rainfall) explained 88% of the variation in dengue incidence rate compared to the best fit model which explained 92% variation. Additionally, AIC decreased substantially from 8970.655 to 4895.068 after including Nino3.4 and DMI into the model. There was no significant auto-correlation between model residuals at different lags in the DLNM (Supplementary Figure S8). Thus the goodness-of-fit analyses show that the model fits the data reasonably well. The cross validation indicates that the DLNM had reasonable accuracy as the observed and predicted values were mostly consistent (Fig. 4). Thus although neither Nino3.4 nor DMI was significantly associated with dengue incidence in the partial correlation analysis, they became significant in the DLNM after adjustment for local climate variables, seasonality and trend. The coefficients of the best fit model are presented in Table 4. In the best fit model, the coefficients for Nino3.4 were 8.2 (*p* < 0.01) and 7.6 for DMI (*p* = 0.01) (Table 4). Figure 5 illustrates the overall association between Nino3.4 and DMI with dengue incidence in the final model. The estimated effects were nonlinear for both Nino3.4 and DMI with higher relative risks at higher Nino3.4 and DMI (Fig. 5). For example, the higher Nino3.4 index was associated with higher dengue incidence at 4-month lag. We calculated the overall effects of Nino3.4 and DMI on dengue incidence. A unit increase (0.1) in Nino3.4 (reference value = −0.1) and DMI (reference value = 0.1) was associated with a 3.3% (95% confidence interval (CI): 0.8, 12.3) and 2.5% (95% CI: 0.5, 11.7) increase in dengue incidence, respectively (Table 5).

## Discussion

We found that ENSO and IOD might play a role in the inter-annual variation in dengue incidence in Bangladesh. However, the association was very weak and might be driven by correlation between dengue incidence and local climate variables such as temperature and rainfall. The ENSO has been reported to be a driver of the inter-annual variation of endemic dengue in many parts of the world^5^,^18^,^19^,^20^,^21^. The biological basis for this relationship is that ENSO brings changes in local weather, and local weather affects dengue transmission. In Bangladesh, higher ENSO was associated with higher dengue incidence. Winter ENSO events lead to a general warming of the tropical atmosphere that persists into the next summer, potentially extending the breeding season for mosquitoes and their spatial distribution. This warming leads to a change in the circulation over the Indian Ocean region, which brings greater moisture and increases monsoon rainfall over Bangladesh^12^. Hales and his colleagues examined the relationship between ENSO and monthly incidence of dengue in 14 island nations of the Pacific^5^. They found that dengue incidence was positively associated with ENSO in 10 countries, which is consistent with our findings. Studies in Thailand and Mexico also reported a positive association between dengue and ENSO^18^,^22^. Similar relationship between dengue and ENSO was observed in Australia^19^. Australia is likely to experience dry and warmer than normal conditions when El Niño occurs. Although drier conditions may reduce breeding sites this is likely for the container breeding mosquitoes such as *Ae. aegypti and Ae. albopictus*. However, warmer temperatures allow for increased mosquito reproduction and activity and more rapid larval development which results in an increased capacity for producing offspring^19^.

Using wavelet coherency analysis, we found very weak associations of ENSO and IOD with dengue at a multiyear scale. There was a weak coherence between ENSO and dengue at a periodicity of 1–2 years, which did not reach statistical significance. The coherence between IOD and dengue was observed at a periodicity of 1 year. ENSO and IOD affect local weather conditions which in turn affect dengue incidence in Bangladesh. There is also evidence of a nonstationary relationship between ENSO and dengue incidence in the 2–3 year periodic cycle in Thailand and Vietnam^4^,^23^. Johansson and his colleagues also observed a weak associations between dengue and ENSO in Puerto Rico, Thailand and Mexico^24^. The role of ENSO may be masked by local variation in climate, randomly coincident outbreaks and other factors regulating dengue dynamics such as socio-ecological conditions and vector control.

In the DLNM, we observed a positive association between IOD and dengue after controlling for local climate variables, seasonality and trend. During a positive IOD event high rainfall occurs over Bangladesh, which increases the number of breeding habitats for *Aedes* mosquitoes. Rainfall has previously been reported to influence dengue in Bangladesh^13^. There is also evidence that IOD has influenced the cholera incidence in Bangladesh^25^,^26^. The key characteristics of dengue incidence included a relationship with ENSO and IOD in the preceding 1–6 months. This delay is biologically plausible as dengue transmission requires time for mosquito and virus development with external incubation (within mosquito) and internal incubation (within host). This feature may be used to assist in forecasting dengue outbreaks in Bangladesh. Four months lag effect of ENSO was observed in Mexico^22^ and a longer lag (1–6 month) was observed in Thailand^18^.

We observed a broad confidence interval for the association between dengue incidence and ENSO or DMI. This may be due to the relatively shorter study period with limited observations (154 months). The first dengue outbreak in Bangladesh occurred in 2000. A longer time period may improve our model and narrow the confidence interval. Notwithstanding this, our 154 month dataset adequately captured the trend and seasonality in the dataset. Of course, the use of weekly dengue notification data rather than monthly data would increase the number of observations but both ENSO and IOD are long term global climate phenomena so weekly data could be too small to capture their effects on dengue.

This is the first epidemiological study for Bangladesh to explore the impacts of IOD on dengue incidence and a predictive model was developed for dengue using both IOD and ENSO indices. The model has a high degree of accuracy. We acknowledge the limitations inherent in using passive surveillance data. Dengue data used in this study were based on notified suspected dengue cases from hospitals and clinics without laboratory confirmation. Underreporting bias is possible in dengue surveillance when people infected with subclinical dengue infection or did not seek medical advice^19^. We did not adjust our model for the effects of population immunity, mosquito density, viral factors and human behaviour as these data are unavailable. More detailed risk assessment at a more local or individual level may improve the model.

Although the findings of this study cannot be interpreted as a causal relationship, they do suggest that inclusion of ENSO and IOD could improve the model fitness when building predictive models for dengue, if more sophisticated models are employed to capture nonlinear trends and take appropriate account of other factors. This study furthers our understanding of our ability to predict dengue epidemics in Bangladesh. An accurate and precise predictive model will allow for recognition of epidemic periods at an early stage, thereby facilitating intervention with an adequate 1–6 month lead time. Early warning using both local and long-term variables can assist in improving vector control and personal protection and provide a useful decision support tool for dengue risk management.

## Methods

### Data collection

The data consist of a monthly time series of dengue incidence in Bangladesh from January 2000 to October 2012 provided by the Directorate General of Health Services (DGHS), Bangladesh. This data is freely available through formal data request in DGHS. The ENSO index used in this study is the Nino3.4 (SST anomaly index for the Nino region 3.4 available from the climate prediction centre of the US National Weather Service (http://www.cpc.ncep.noaa.gov/data/indices). The monthly average DMI values were obtained from the Japan Agency for Marine Earth Science and Technology http://www.jamstec.go.jp/frsgc/research/d1/iod/e/iod/dipole_mode_index.html). Over the same time span, the monthly weather data on temperature, relative humidity and total rainfall were obtained from 35 weather stations in Bangladesh. Population data (2001 and 2011 censuses) were obtained from Bangladesh Bureau of Statistics (BBS). The population between censuses were estimated using a linear interpolation approach.

### Data analysis

#### Wavelet coherence analysis

Wavelet analysis provides the possibility of investigating and quantifying the temporal evolution of time series with different rhythmic components. It can be used to determine whether the presence of a particular periodic cycle at a given time in a disease incidence corresponds to the presence of the same periodical cycle at the same time in an exposure covariates^27^. Here we used wavelet coherence analysis to determine whether the presence of a particular frequency at a given time in dengue incidence corresponded to the presence of the same frequency climate covariates such as ENSO and DMI. The Fourier squared coherency is used to identify frequency bands within which two time series are co-varying. The 95% significance level was determined from a Monte Carlo simulation of 300 sets of two white noise time series with the same length as dengue cases and climate variables. Analyses were performed using R package “biwavelet”.

#### Distributed Lag Non-linear Model

A Poisson time series model combined with the DLNM was used to estimate the effects of ENSO and DMI on dengue incidence^16^,^17^. The observed number of dengue cases followed a quasi-Poisson distribution and the model allows for over dispersion.


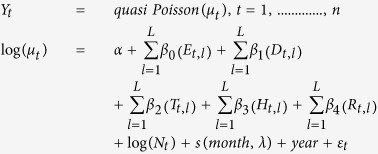


Where *t* is the month of the observation; *Y*_*t*_ is the observed monthly dengue counts in month *t* which implies logarithm of the expected monthly dengue cases μ_t_; log(μ_t_) is the logarithm of the expected monthly dengue incidence μ_t_ ; α is the intercept; *E*_*t,l*,_*D*_*t,l*,_*T*_*t,l*,_ *H*_*t,l*_ and *R*_*t,l*_ are the matrices obtained by applying the DLNM to ENSO, DMI, temperature, humidity and rainfall, respectively; *l* is the lag months; L is the maximum lag; *β*_*0*_, *β*_*1*,_ *β*_*2*,_ *β*_*3*_ and *β*_*4*_ are the vector coefficients for *E*_*t,l*,_*D*_*t,l*,_*T*_*t,l*,_ *H*_*t,l*_ and *R*_*t,l*_ respectively; *N*_*t*_ is an offset to control for population using a linear function of time based on the 2001 and 2011 censuses. The *s(month, λ)* is the natural cubic spline smoothing function of calendar month with assigned λ of 6 degrees of freedom to control for seasonal pattern and trend. To take account of the time taken for mosquitoes to lay eggs, hatch and develop to adults and for the virus to replicate, we incorporated time lags into the analysis. We used a natural cubic spline basis with three degrees of freedom for ENSO and two degrees of freedom for lag. A natural cubic spline with three degrees of freedom was used for both DMI and the lag. We adjusted the model for temperature and humidity with a maximum lag of four months. We also adjusted for rainfall using natural cubic spline with a maximum lag of six months. We used the maximum lag up to 6 months according to previous study^22^,^28^ and the selection of maximum lag was conducted using model residual checking. We checked the auto-correlation function of the residuals and the normality of the residuals. To choose the degrees of freedom, we used the Akaike Information Criterion (AIC) for quasi-Poisson models. The median value was defined as the baseline (reference value) for calculating the relative risks. We plotted relative risks against ENSO and DMI to show the entire relationship between weather conditions and dengue. The constructed model was then validated using cross validation method. 2011 and 2012 data were removed to create the testing dataset and the remaining dataset was used as training dataset. The validation was run and then the predicted values were plotted against the observed values Analyses were performed using R package “dlnm”^29^.

## Additional Information

**How to cite this article**: Banu, S. *et al.* Impacts of El Niño Southern Oscillation and Indian Ocean Dipole on dengue incidence in Bangladesh. *Sci. Rep.* **5**, 16105; doi: 10.1038/srep16105 (2015).

## Supplementary Material

Supplementary Information

## Figures and Tables

**Table 1:** Descriptive statistics for monthly climate variables and monthly dengue incidence rate in Bangladesh.

Variable	Mean	Standard Deviation	Minimum	Maximum
Incidence rate (1/100,000)	0.13	0.32	0	2.56
Temperature (^o^C)	25.5	3.8	16.3	29.7
Relative Humidity (%)	79.85	5.3	67	88
Rainfall (mm)	7086	7104	0	24638
Nino3.4	−0.1	0.75	−1.79	1.79
DMI	0.12	0.57	−1.27	1.37

**Table 2:** Partial correlations between monthly climate variables and dengue incidence rate in Bangladesh.

Variables	Incidence rate	Temperature	Humidity	Rainfall	DMI
Temperature	0.09				
Humidity	0.26*	−0.15			
Rainfall	−0.07	0.57*	0.7*		
DMI	0.02	0.12	0.07	−0.04	
Nino3.4	0.07	0.02	0.09	−0.35	0.29*

^*^P < 0.01.

**Table 3:** Adjusted R2 and Akaike Information Criterion (AIC) for different distributed lag nonlinear models (DLNM).

DLNMs	Adjusted R2	AIC
Nino3.4	0.562	20480.45
DMI	0.77	14016.75
Temperature	0.596	20229.21
Humidity	0.743	17886.6
Rainfall	0.833	11147
Temperature, Humidity, Rainfall	0.88	8970.655
Temperature, Humidity, Rainfall and Nino3.4	0.902	7812.226
Temperature, Humidity, Rainfall and DMI	0.908	6912.696
Temperature, Humidity, Rainfall, Nino3.4 and DMI	0.929	4895.068

**Table 4:** Coefficients of the best fit distributed lag nonlinear model.

Variables	df	F	p
ENSO	6	8.2	2.1e***
DMI	9	7.6	1.07e***
Temperature	2	3.9	0.02**
Humidity	2	9.2	0.00**
Rainfall	8	9.5	4.7e***
Year	1	26.8	9.47e***
s(month,6)	5	6.2	3.7e***

**Table 5:** Association between dengue incidence and DMI and ENSO in the best fit model.

Variables	Reference values	Estimates	95% confidence interval
0.1 increase in Nino3.4	−0.1	3.3	0.8, 12.3
0.1 increase in DMI	0.1	2.5	0.5, 11.7

## References

[b1] Gubler D. J. Dengue and dengue hemorrhagic fever. Clin. Microbiol. Rev. 11, 480–496, (1998). 9665979 10.1128/cmr.11.3.480 PMC88892

[b2] Hopp M. J. & Foley J. A. Global-scale relationships between climate and the dengue fever vector, *Aedes aegypti*. Clim. Chang. 48, 441–463, (2001).

[b3] Patz J. A., Martens W. J. M., Focks D. A. & Jetten T. H. Dengue fever epidemic potential as projected by general circulation models of global climate change. Environ. Health Perspect. 106, 147–153, (1998). 9452414 10.1289/ehp.98106147 PMC1533051

[b4] Cazelles B., Chavez M., McMichael A. J. & Hales S. Nonstationary influence of EI Nino on the synchronous dengue epidemics in Thailand. PLoS Med. 2, 313–318, (2005). 10.1371/journal.pmed.0020106 PMC1087219 15839751

[b5] Hales S., Weinstein P., Souares Y. & Woodward A. El Nino and the dynamics of vector-borne disease transmission. Environ. Health Perspect. 107, 99–102, (1999). 9924003 10.1289/ehp.9910799 PMC1566321

[b6] Kovats R. S., Bouma M. J., Hajat S., Worrall E. & Haines A. El Nino and health. Lancet 362, 1481–1489, (2003). 14602445 10.1016/S0140-6736(03)14695-8

[b7] Kumar K. K., Rajagopalan B., Hoerling M., Bates G. & Cane M. Unraveling the Mystery of Indian Monsoon Failure During El Niño. Science 314, 115–119, (2006). 16959975 10.1126/science.1131152

[b8] Pascual M., Rodo X., Ellner S. P., Colwell R. & Bouma M. J. Cholera dynamics and El Nino-Southern Oscillation. Science 289, 1766–1769, (2000). 10976073 10.1126/science.289.5485.1766

[b9] Hashizume M., Faruque A. S., Wagatsuma Y., Hayashi T. & Armstrong B. Cholera in Bangladesh: climatic components of seasonal variation. Epidemiology 21, 706–710, (2010). 20562706 10.1097/EDE.0b013e3181e5b053

[b10] Saji N. H., Goswami B. N., Vinayachandran P. N. & Yamagata T. A dipole mode in the tropical Indian Ocean. Nature 401, 360–363, (1999). 16862108 10.1038/43854

[b11] Hashizume M., Terao T. & Minakawa N. The Indian Ocean Dipole and malaria risk in the highlands of western Kenya. Proc. Natl. Acad. Sci. USA 106, 1857–1862, (2009). 19174522 10.1073/pnas.0806544106 PMC2644128

[b12] Ashok K., Guan Z., Saji N. H. & Yamagata T. Individual and Combined Influences of ENSO and the Indian Ocean Dipole on the Indian Summer Monsoon. J. Climate 17, 3141–3155, (2004).

[b13] Hashizume M., Dewan A. M., Sunahara T., Rahman M. Z. & Yamamoto T. Hydroclimatological variability and dengue transmission in Dhaka, Bangladesh: a time-series study. BMC Infect. Dis. 12, 98, (2012). 22530873 10.1186/1471-2334-12-98 PMC3528427

[b14] Karim M. N., Munshi S. U., Anwar N. & Alam M. S. Climatic factors influencing dengue cases in Dhaka city: a model for dengue prediction. Indian J. Med. Res. 136, 32–39, (2012). 22885261 PMC3461715

[b15] DGHS. Operational plan: Communicable disease control (2011).

[b16] Gasparrini A., Armstrong B. & Kenward M. Distributed lag non-linear models. Stat. Med. 29, 2224 (2010). 20812303 10.1002/sim.3940 PMC2998707

[b17] Gasparrini A. Modeling exposure–lag–response associations with distributed lag non-linear models. Stat. Med. 33, 881–899 (2014). 24027094 10.1002/sim.5963 PMC4098103

[b18] Tipayamongkholgul M., Fang C. T., Klinchan S., Liu C. M. & King C. C. Effects of the El Nino-Southern Oscillation on dengue epidemics in Thailand, 1996-2005. BMC Public Health 9, 422 (2009). 19930557 10.1186/1471-2458-9-422 PMC2785791

[b19] Hu W., Clements A., Williams G. & Tong S. Dengue fever and El Niño/Southern Oscillation in Queensland, Australia: a time series predictive model. Occup. Environ. Med. 67, 307–311 (2010). 19819860 10.1136/oem.2008.044966

[b20] Brunkard J. M., Cifuentes E. & Rothenberg S. J. Assessing the roles of temperature, precipitation, and ENSO in dengue re-emergence on the Texas-Mexico border region. Salud Publica Mex. 50, 227–234 (2008). 18516370 10.1590/s0036-36342008000300006

[b21] Earnest A., Tan S. B. & Wilder-Smith A. Meteorological factors and El Nino Southern Oscillation are independently associated with dengue infections. Epidemiol. Infect. 140, 1244–1251 (2012). 21906411 10.1017/S095026881100183X

[b22] Hurtado-Diaz M., Riojas-Rodriguez H., Gomez-Dantes H. & Cifuentes E. Short communication: Impact of climate variability on the incidence of dengue in Mexico. Trop. Med. Int. Health 12, 1327–1337 (2007). 17956543 10.1111/j.1365-3156.2007.01930.x

[b23] Thai K. T. *et al.* Dengue dynamics in binh thuan province, southern Vietnam: periodicity, synchronicity and climate variability. PLoS Negl. Trop. Dis. 4, e747 (2010). 20644621 10.1371/journal.pntd.0000747 PMC2903474

[b24] Johansson M. A., Cummings D. A. T. & Glass G. E. Multiyear climate variability and dengue—El Nino Southern Oscillation, weather, and dengue Incidence in Puerto Rico, Mexico, and Thailand: a longitudinal data analysis. PLoS Med. 6, e1000168 (2009). 19918363 10.1371/journal.pmed.1000168 PMC2771282

[b25] Hashizume M. *et al.* The Indian Ocean dipole and cholera incidence in Bangladesh: a time-series analysis. Environ. Health Perspect. 119, 239–244 (2011). 20980219 10.1289/ehp.1002302 PMC3040612

[b26] Hashizume M. *et al.* A Differential Effect of Indian Ocean Dipole and El Niño on Cholera Dynamics in Bangladesh. PLoS One 8, e60001 (2013). 23555861 10.1371/journal.pone.0060001 PMC3612031

[b27] Torrence C. & Compo G. P. A Practical Guide to Wavelet Analysis. Bulletin of the American Meteorological Society 79, 61–78 (1998).

[b28] Banu S., Hu W., Guo Y., Hurst C. & Tong S. Projecting the impact of climate change on dengue transmission in Dhaka, Bangladesh. Environment International 63, 137–142 (2014). 24291765 10.1016/j.envint.2013.11.002

[b29] Gasparrini A. & Armstrong B. Distributed lag non-linear model in R: the package dlnm (2011).<cran.r.project.org/web/packages/dlnm/vignettes/dlnmOverview.pdf>.

