# Supplementary material for: Impacts of El Niño Southern Oscillation and Indian Ocean Dipole on dengue incidence in Bangladesh
Source: Sci Rep. 2015 Nov 5;5:16105. doi: 10.1038/srep16105 (PMC4633589; doi:10.1038/srep16105)
Supplement: Supplementary Information [file srep16105-s1.pdf]

Supplementary information for Impacts of El Niño Southern Oscillation and Indian Ocean dipole on dengue incidence in Bangladesh

Shahera Banu[1*], Yuming Guo[2], Wenbiao Hu[1], Pat Dale[3], John S. Mackenzie[4], Kerrie Mengersen[5] and Shilu Tong[1]

1. School of Public Health and Social Work, Queensland University of Technology, Brisbane, QLD 4059, Australia.

2. School of Population Health, University of Queensland, Brisbane, QLD 4006, Australia.

3. Environmental Futures Research Institute, Griffith School of Environment, Griffith University, Brisbane, QLD 4111, Australia

4. Faculty of Health Sciences, Curtin University, GPO Box U1987,Perth, WA 6845, Australia

5 School of Mathematical Science, Queensland University of Technology, Brisbane, QLD 4059, Australia

*****To whom correspondence should be addressed. Email: s.banu@qut.edu.au

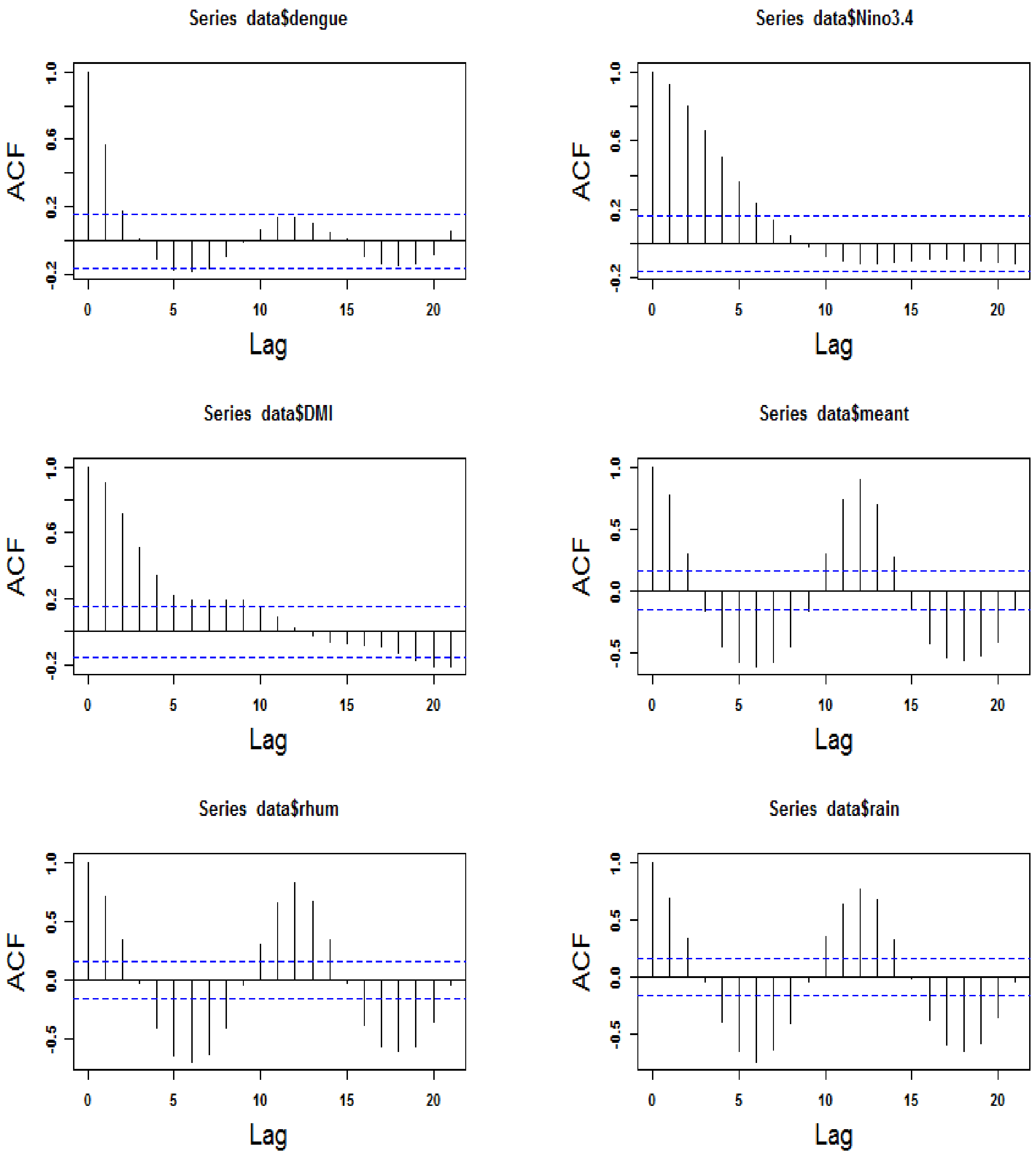

Figure S1: Auto-correlation functions for dengue and climate variables.

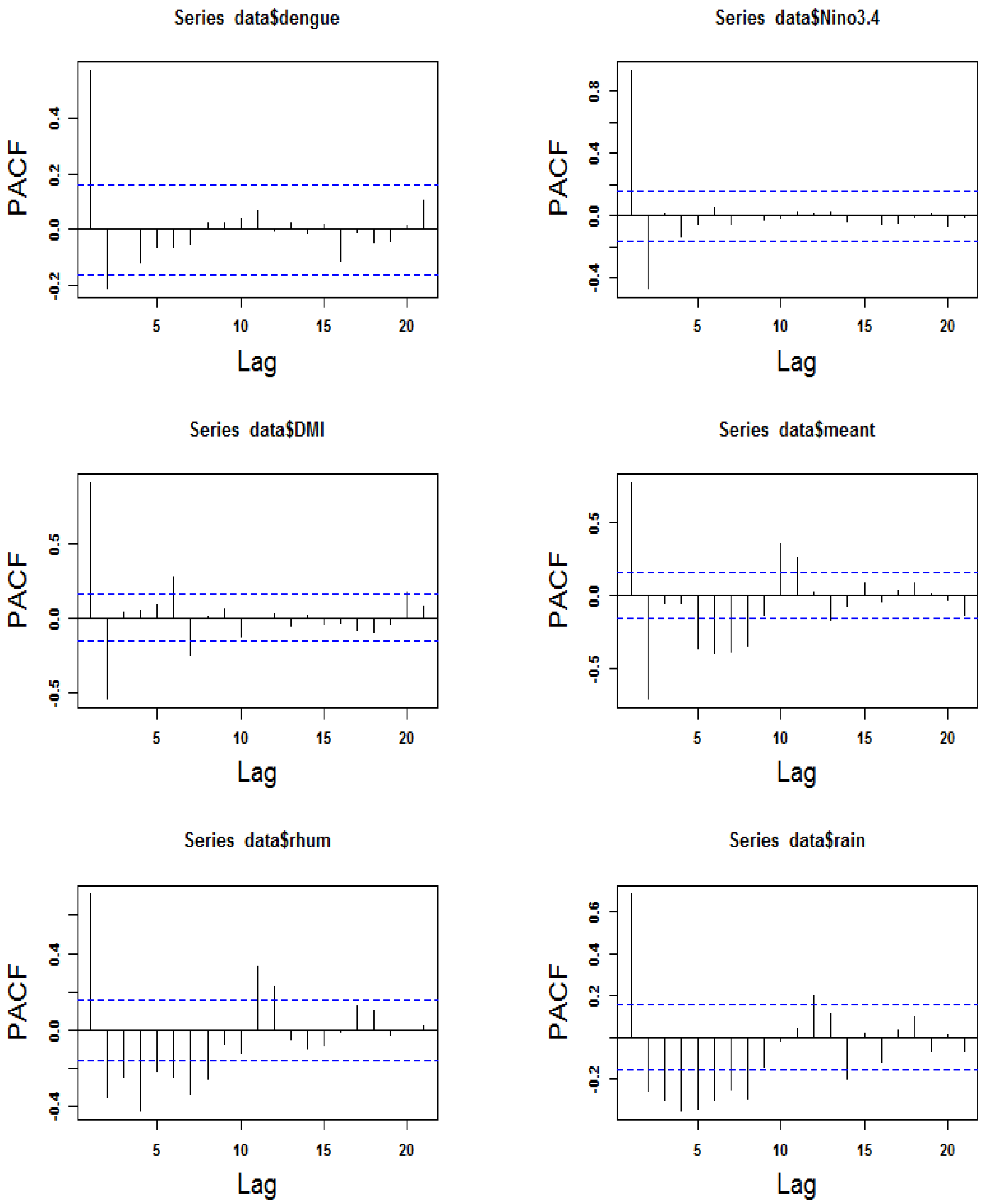

Figure S2: partial autocorrelation functions for dengue and climate variables.

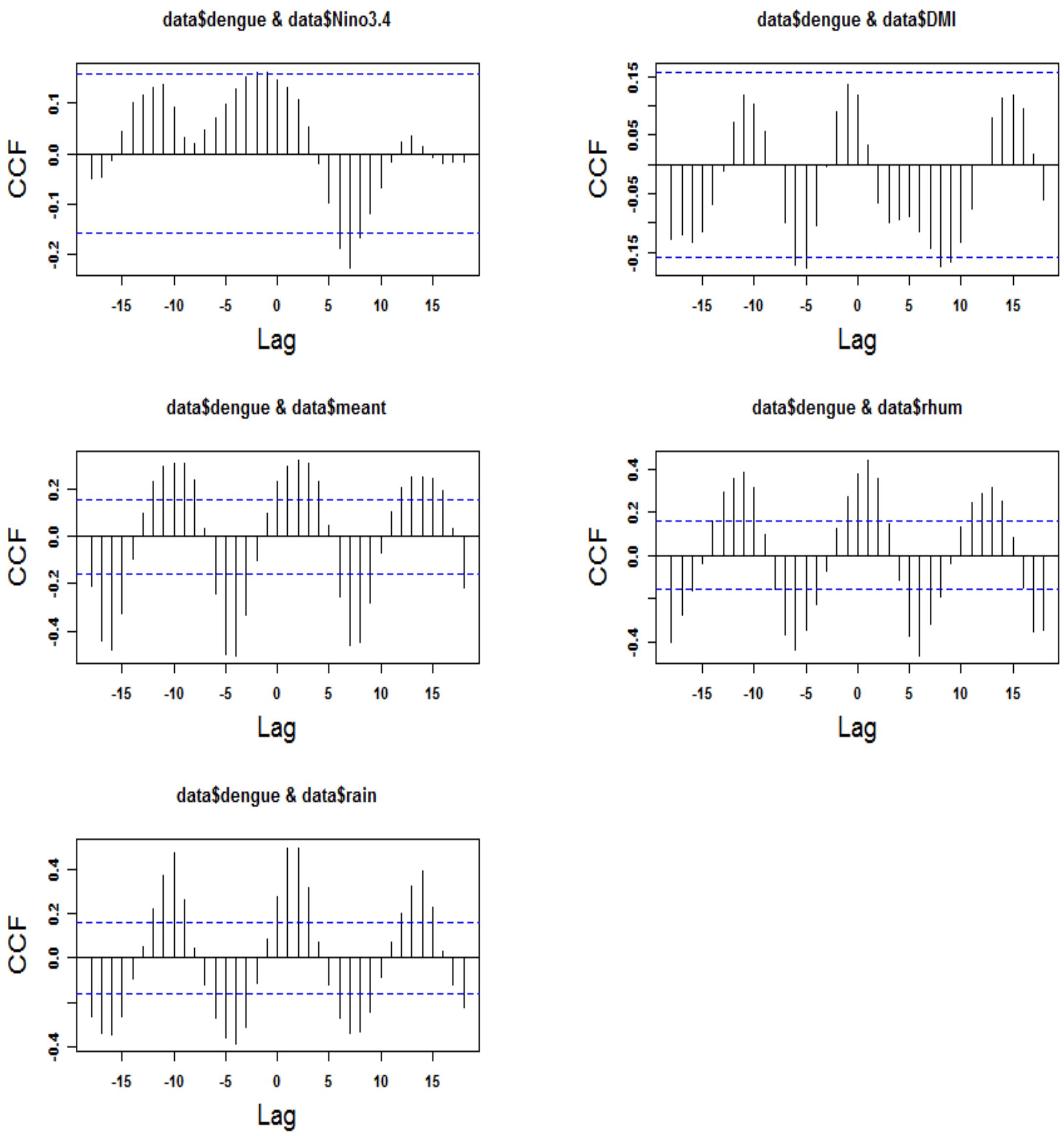

Figure S3: Cross correlation function between dengue and climate variables.

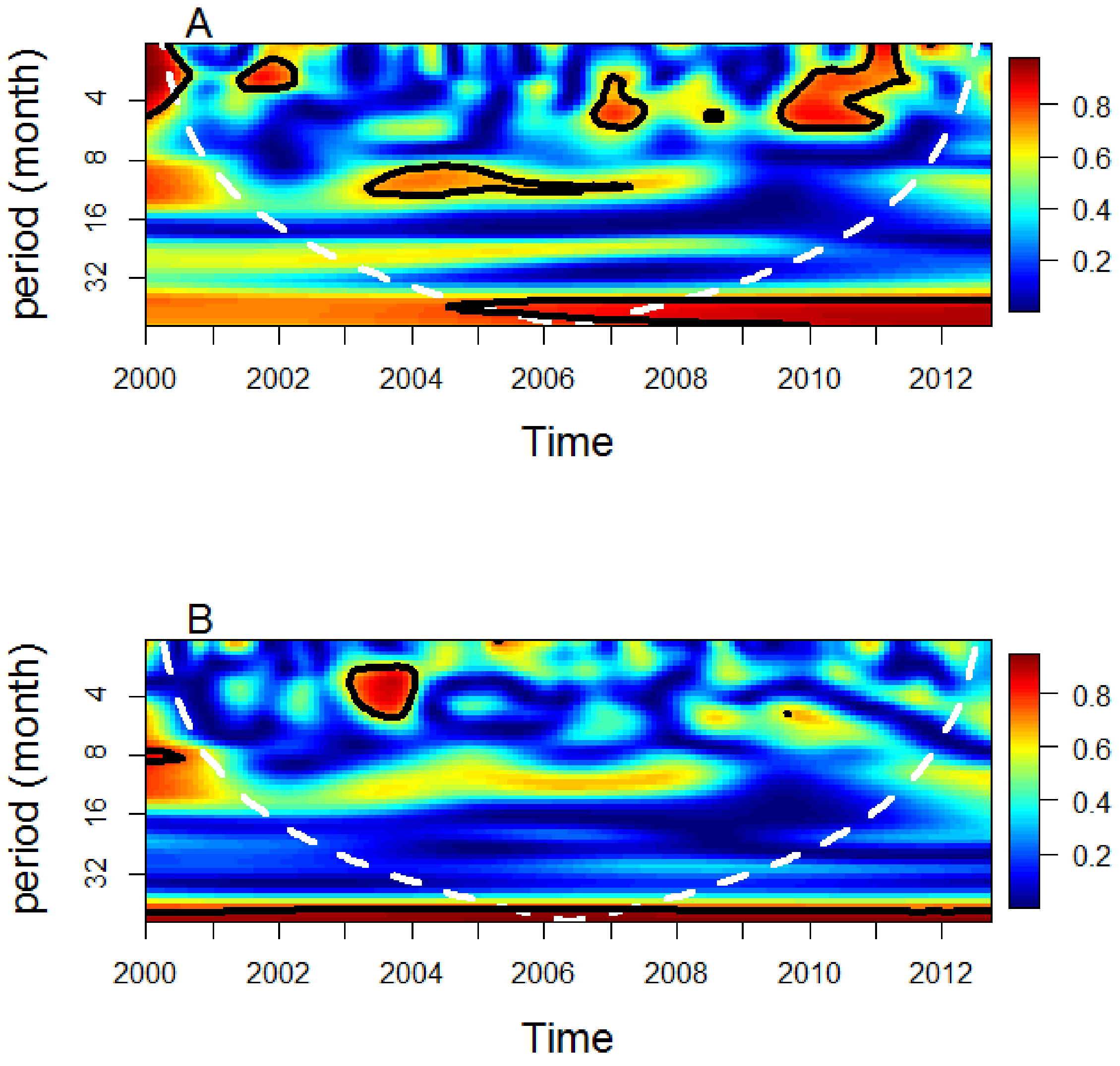

Figure S4: Coherence between Nino3.4 and local climate variables (A: Temperature; B: Rainfall)

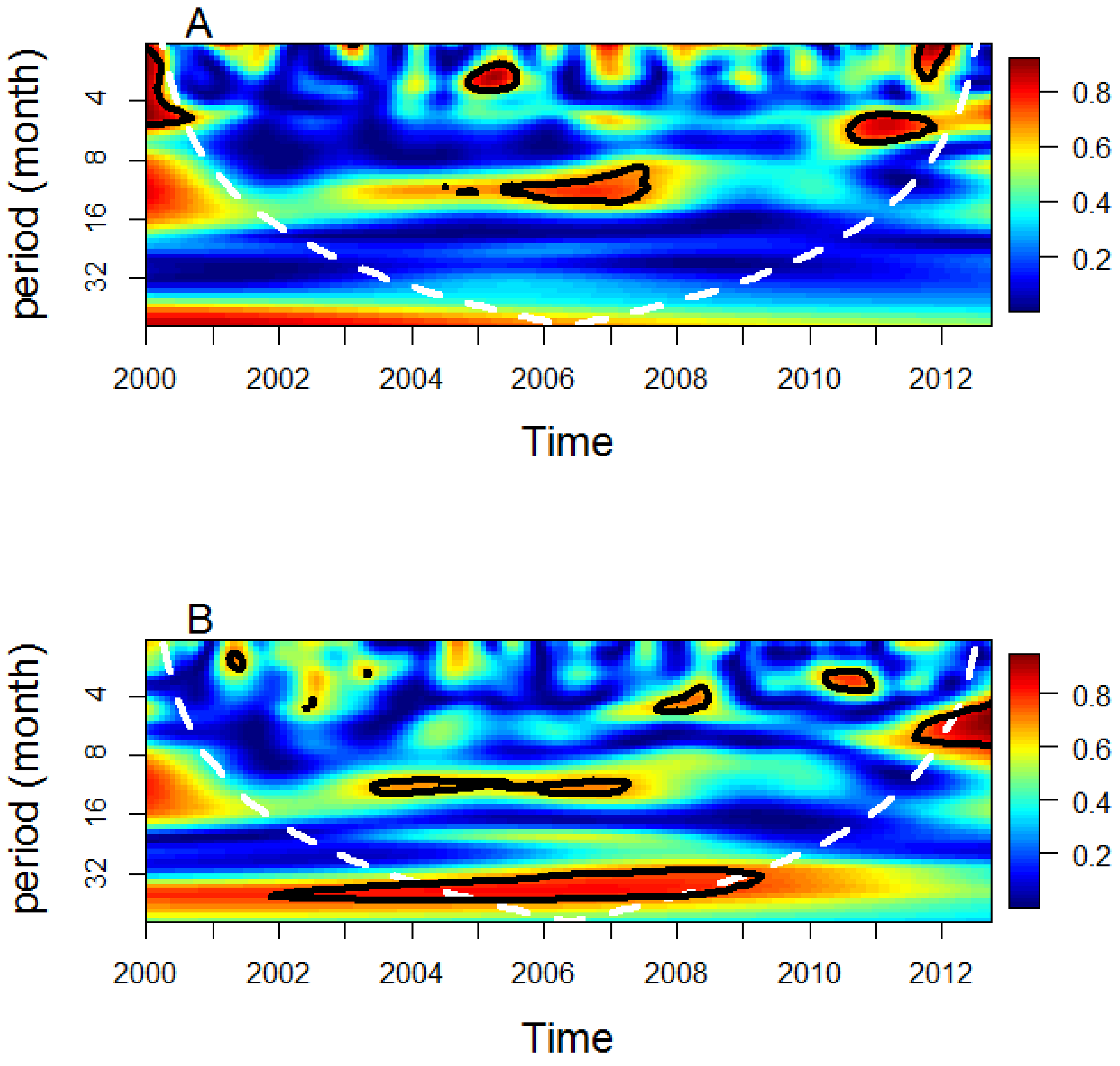

Figure S5: Coherence between DMI and local climate variables (A: Temperature; B: Rainfall).

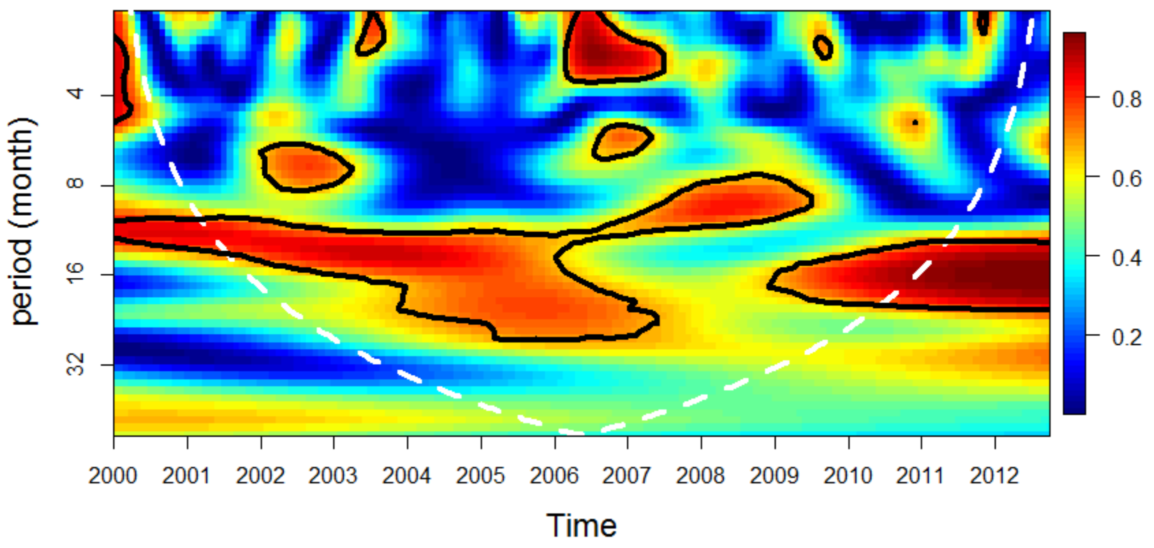

Figure S6: Coherence between Nino3.4 and DMI.

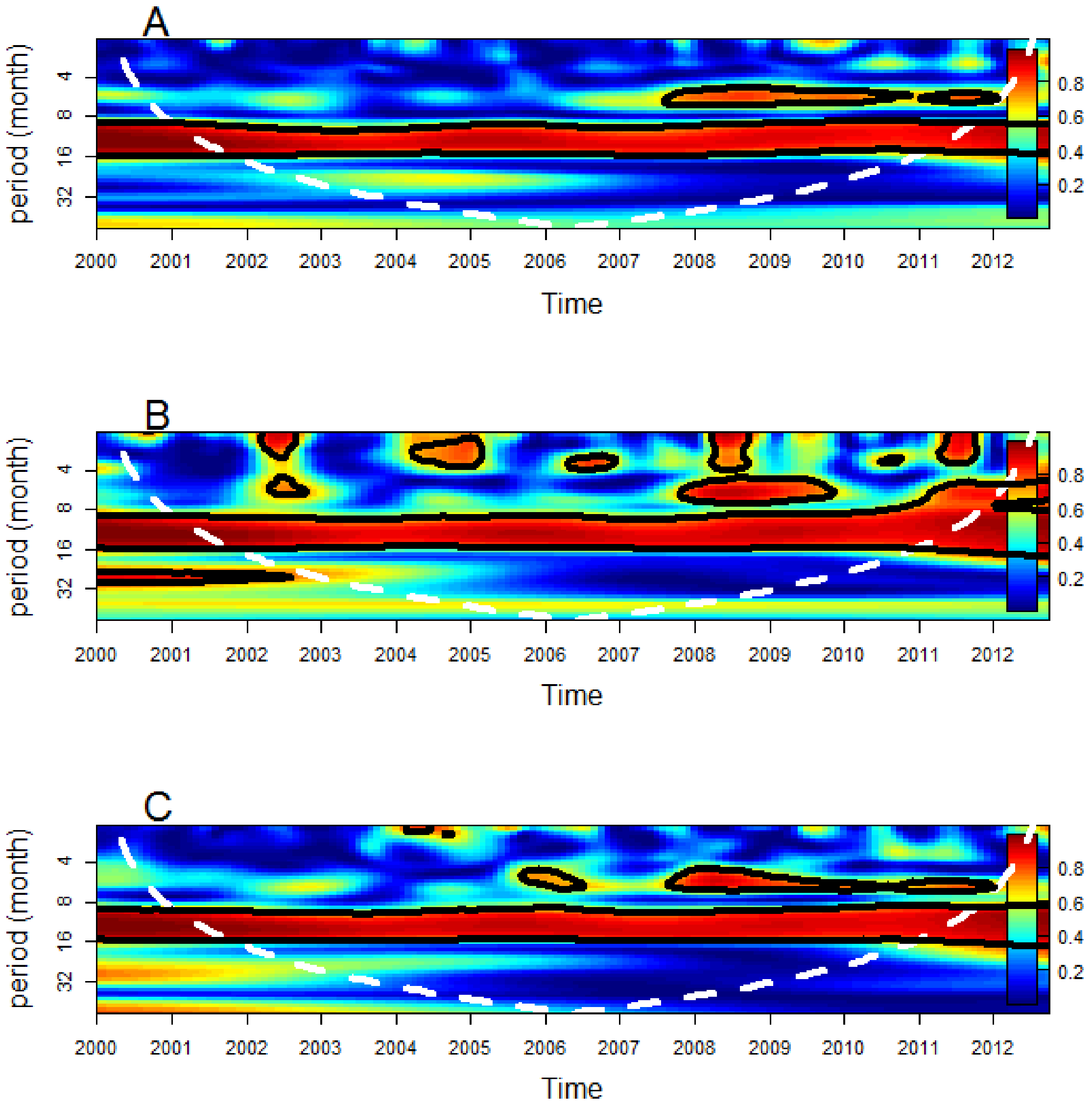

Figure S7: Coherence between dengue incidence and local climate variables (A: Temperature; B: Rainfall; C: Humidity)

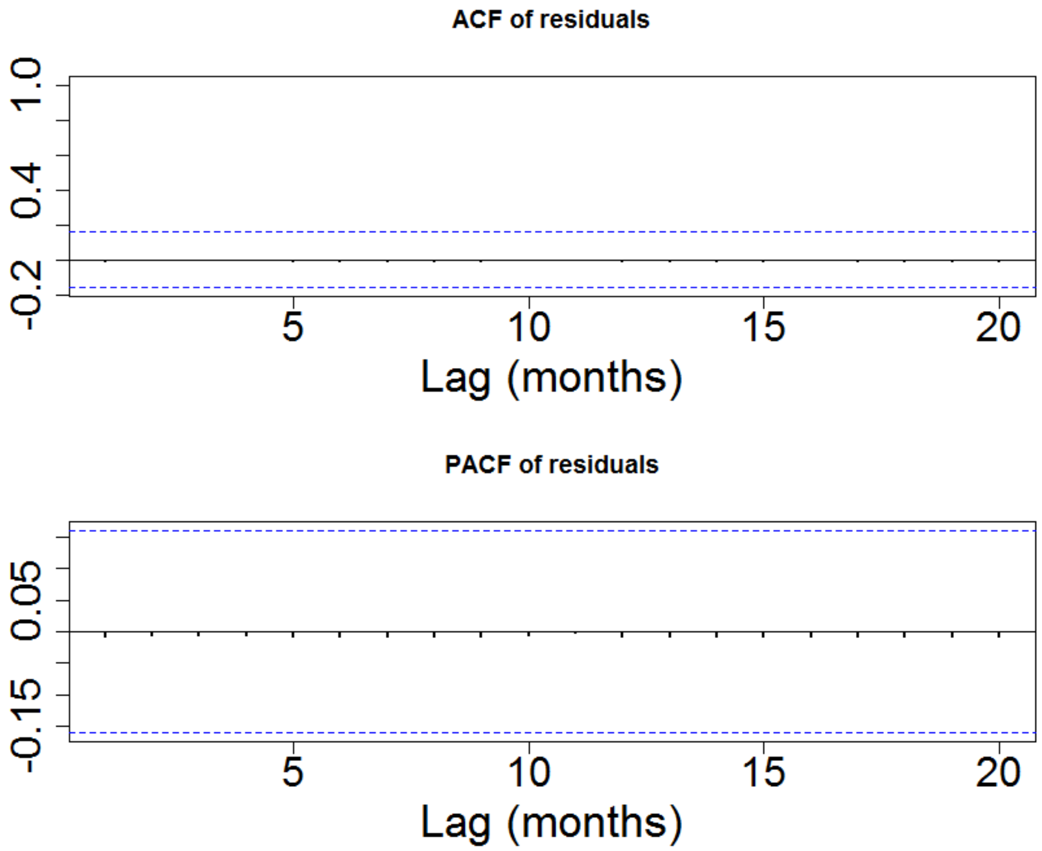

Figure S8: Residual checks for regression model (auto-correlation and partial autocorrelation functions for residuals).
